# Becoming a parent to a child with birth asphyxia—From a traumatic delivery to living with the experience at home

**DOI:** 10.3402/qhw.v8i0.20539

**Published:** 2013-04-30

**Authors:** Alina Heringhaus, Michaela Dellenmark Blom, Helena Wigert

**Affiliations:** 1 Queen Silvia Children's Hospital, Sahlgrenska University Hospital, Gothenburg, Sweden; 2 Sahlgrenska Academy at the University of Gothenburg

**Keywords:** Hypothermia treatment, neonatal care, nursing, infant, traumatic delivery, qualitative content analysis

## Abstract

The aim of this study is to describe the experiences of becoming a parent to a child with birth asphyxia treated with hypothermia in the neonatal intensive care unit (NICU). In line with the medical advances, the survival of critically ill infants with increased risk of morbidity is increasing. Children who survive birth asphyxia are at a higher risk of functional impairments, cerebral palsy (CP), or impaired vision and hearing. Since 2006, hypothermia treatment following birth asphyxia is used in many of the Swedish neonatal units to reduce the risk of brain injury. To date, research on the experience of parenthood of the child with birth asphyxia is sparse. To improve today's neonatal care delivery, health-care providers need to better understand the experiences of becoming a parent to a child with birth asphyxia. A total of 26 parents of 16 children with birth asphyxia treated with hypothermia in a Swedish NICU were interviewed. The transcribed interview texts were analysed according to a qualitative latent content analysis. We found that the experience of becoming a parent to a child with birth asphyxia treated with hypothermia at the NICU was a strenuous journey of overriding an emotional rollercoaster, that is, from being thrown into a chaotic situation which started with a traumatic delivery to later processing the difficult situation of believing the child might not survive or was to be seriously affected by the asphyxia. The prolonged parent–infant separation due to the hypothermia treatment and parents’ fear of touching the infant because of the high-tech equipment seemed to hamper the parent–infant bonding. The adaption of the everyday life at home seemed to be facilitated by the follow-up information of the doctor after discharge. The results of this study underline the importance of family-centered support during and also after the NICU discharge.

In line with the medical advances, the survival of critically ill infants with increased risk of morbidity is increasing (Ehrenstein et al., 2009), which is also the case of infants with birth asphyxia. About one to two infants per 1000 suffer from perinatal asphyxia and neonatal deaths due to perinatal asphyxia are globally estimated to equal 1 million (23%) each year (Lawn, Cousens, & Zupan, 2005). Children who survive birth asphyxia are at a higher risk of functional impairments, cerebral palsy (CP), or impaired vision and hearing (Battin, Dezoete, Gunn, Gluckman, & Gunn, 2001; Ehrenstein et al., 2009). During birth, asphyxia occurs when the child suffers a combination of oxygen deficiency and reduced blood supply that can be indicated by an Apgar score at 10 min<5 points or the pH of the blood is<7.0 (Azzopardi, 2010). In serious cases of asphyxia, the infant shortly after birth can develop symptoms of hypoxic ischemic encephalopathy (HIE). In moderate to severe asphyxia, the lack of oxygen can cause injury to the brain and also to other organs, and some of these critically ill infants die (Azzopardi, 2010; Battin et al., 2001).

In birth asphyxia, the infant needs immediate professional care in the neonatal intensive care unit (NICU; Long & Brandon, 2007), and hypothermia treatment is a method for treating HIE following birth asphyxia (Shankaran & Laptook, 2007) to reduce the risk of brain injury (Yager, Armstrong, Jaharus, Saucier, & Wirrell, 2004). Hypothermia treatment was introduced in 2005 in several neonatal units in various countries that participated in Total Body Cooling trial (TOBY), an English multi-center study (Azzopardi et al., 2009). Medical studies of hypothermia treatment (Blackmon & Stark, 2006; Edwards & Azzopardi, 2006; Perlman, 2006) was based on the specified National Guidelines for the Medical Hypothermia Care in Sweden. Since the end of 2006, hypothermia treatment following birth asphyxia is used in many of the Swedish neonatal units (Swedish Paediatric Society, 2005), including the NICU specifically chosen in this study.

Hypothermia treatment of the newborn involves cooling the infant for a period of 72 h to a body temperature of 33°C–34°C. Then, the infant's body temperature is increased by 0.5°C per hour until the normal body temperature (37°C±0.2°C) is reached (Groenendaal & Brouwer, 2009). The infant is given both pain medication and sedation using a technical monitoring apparatus. Light, sound, and manipulation is kept minimal to prevent the rise of pressure in the brain. All nursing actions and investigations are well planned and coordinated for the infant to be disturbed as little as possible. Due to the fragile medical condition and the infant's need of calmness, it is usually not possible for parents to hold the infant during hypothermia treatment (Long & Brandon, 2007). Predicting the outcome during the first days of the hypothermia treatment is very difficult, but after completing the hypothermia treatment, a magnetic resonance imaging (MRI) is carried out to discover any possible brain injuries (Roka & Azzopardi, 2010). These specific circumstances may possibly affect the experience of becoming a parent and the establishment of the parent–infant relationship. However, research on this particular topic is sparse. Previous researches show that parents’ experience of having a premature or a critically ill infant at the NICU is associated with stress, fear, worry, and guilty (Arockiasamy, Holsti, & Albersheim, 2008; Heermann, Wilson, & Wilhelm, 2005; Lefkowitz, Baxt, & Evans, 2010; Pinelli et al., 2008; Wigert, Johansson, Berg, & Hellström, 2006) and that there is a correlation between the experience of the delivery as a traumatic event and posttraumatic stress reactions (Beck, 2004). In the NICU, parents can find the environment unfamiliar and Hall (2005) even stated that the parents experienced the NICU as an alien world. Being unknown of the infant's future condition is a difficult experience. Parents are coping up with the situation more easily when they receive clear and frequent information about the infant's condition and have explanations about the department's routines as well as the purpose of the equipment used for monitoring the infant's health (Arockiasamy et al., 2008; Heermann et al., 2005; Wigert et al., 2006). However, a study by Wigert, Berg, and Hellström (2007) showed that health-care professionals in the NICU needed further improvements to correctly deal with the emotional crisis of parents and involving them in the infant's care.

To date, research on the experience of parenthood of the child with birth asphyxia is sparse. In a qualitative exploratory study by Nassef, Blennow, and Jirwe (2013), the authors found that parents, whose infant undergoes hypothermia treatment following perinatal asphyxia, used several strategies to cope up with their stress during the NICU time, and that they needed nursing support in processing their experiences. The authors also concluded that further research is warranted to provide the correct support to the families whose infants undergo the hypothermia treatment (Nassef et al., 2013). Subsequently, to improve today's neonatal care delivery, health-care providers need to better understand the experiences of becoming a parent to a child with birth asphyxia. By describing parents’ experiences of becoming a parent to a child with birth asphyxia, circumstances affecting the establishment of the family and appropriate means of supporting the family will hopefully be more clearly described. Parents of children with neuropsychological impairments are at a greater risk of higher stress levels after NICU (Brummelte, Grunau, Synnes, Whitfield, & Petrie-Thomas, 2011; Iversen, Graue, & Råheim, 2013; Treyvaud et al., 2011) and mothers feelings of ambivalence or alienation in relation to the infant, caused by the separation when the infant needs professional care in the NICU, can persist for several years (Jackson, Ternestedt, & Schollin, 2003; Wigert et al., 2006). This further underlines the need to research the experiences of becoming a parent to a child with birth asphyxia treated with hypothermia at the NICU.

## Aim

The aim of this study is to describe the experiences of becoming a parent to a child with birth asphyxia treated with hypothermia at the NICU.

## Method

### Design

Since the intention was to collect more data about the experiences of becoming a parent to a child with birth asphyxia, a sparsely researched area to date, the authors chose to collect data based on open-ended interviews. The starting point was human communication and interactive sessions, which are effective means to get access to other people's experiences (Merlau-Ponty, 1995/1945). Furthermore, the interview also provides the researcher opportunities to ask the interviewee more explanations and narration (Lindseth & Norberg, 2004).

This study was carried out in accordance with qualitative latent content analysis described by Graneheim and Lundman (2004). Depending on the method chosen, qualitative analysis can be applied to various levels of interpretation. This study is a descriptive qualitative study, and as maintained by Sandelowski (2010), the content analysis is a method that can produce more “data-near” results, than studies within traditions of a deeper interpretation, such as grounded theory and phenomenology. Still, the qualitative latent analysis is based on data from narratives and observations, and deals with the context of the text; therefore, it involves an interpretation and thus produces interpretive products (Graneheim & Lundman, 2004; Sandelowski, 2010). The authors’ choice of qualitative latent content analysis is subsequently derived from the choice of interpretation level.

In the qualitative latent content analysis described by Graneheim and Lundman (2004), the transcribed interview texts, read completely several times, are interpreted step-by-step. First, the analysis began by finding the meaning units, that is, the constellation of words or statements that relate to the same central meaning, such as words, sentences, or paragraphs containing aspects related to each other through their content. Second, the meaning units are condensed, that is, the process of shortening while still preserving the core. Third, these codes are the basis of finding themes or categories. A theme is defined as a thread of an underlying meaning in the text through condensed meaning units or codes. A theme answers the question “How?” and can be constructed by subthemes or divided into subthemes (Graneheim & Lundman, 2004).

### Setting

This study was conducted at an NICU in a university hospital in Sweden that treats about 1000 newborns per year, including extremely premature or critically ill infants from other hospitals in the region. The NICU has two intensive care rooms and two rooms for intermediate care with five to six beds per room, and a maximum of 22 beds. There are two family rooms at the NICU and the majority of parents stay either in the maternity ward, which is located in the same building as the NICU, or in their own home. Some of the parents from a greater distance may stay in a patient hotel close to the children's hospital. In the NICU, where this study was conducted, hypothermia treatment following birth asphyxia has been used since the end of 2006.

### Participants

Since December 2006 to May 2010, 36 children received hypothermia treatment at the NICU. The sample of parents was selected according to the following inclusion criteria: the infant had a verified birth asphyxia treated with hypothermia at the NICU, the infant had survived the asphyxia and the parents could speak and understand the Swedish language. Of the families who matched the criteria, 15 families were excluded due to the following reasons: in five families, the children had died and one family could not speak or understand Swedish. Moreover, nine families lived in a different locality and were not available to contact.

The remaining 21 families were contacted and invited to participate in this study and 16 of them agreed (76%). Of the 32 parents in those 16 families, 26 parents were interviewed. The age of the mothers ranged from 29 to 46 (mean: 36.2 years) and of the fathers is 33–62 (mean: 34.9 years). There were 12 first-time parents (46.2%), and five of the interviewed parents (19.2%) had earlier NICU experiences. In 14 (87.5%) of the interviewed families, the parents were living together, and in two of them (12.5%) they were separated or divorced. The children were born full-term, and the gestational period ranged from 38 to 42 weeks (mean: 40.1 weeks). The birth weight ranged from 2960 to 4700 g (mean: 3688 g). Eight of the children were delivered by caesarean (50.0%), five by vacuum extraction (31.3%), and three of them vaginally without vacuum extraction (18.7%). The children were admitted to the hospital for 7–30 days (mean: 15.6 days). On the day of the interview, the children were aged between 5 months and 4 years. Five of the children were younger than 2 years of age (31.2%), nine of them were between 2 and 3 years (56.2%), and two of them were 4 years (12.5%). Three of the children had persistent injuries (18.8%), such as impaired hearing, facial paresis, impaired sight, motor impairment, or muscular problems.

### Data collection

Information about this study was sent to the parents’ home during August 2010. Shortly after this, the first author contacted the family through telephone and asked whether they are interested to participate in this study. The parents themselves decided on how they wanted to be interviewed. Two of the interviews were conducted in the parents’ working place and the rest in the parents’ home; all of the parents were interviewed by the first author. Ten parents were interviewed as a couple (*n*=10), five couples one by one (*n*=10), and for six couples, either the mother (*n*=5) or father (*n*=1) was interviewed, totaling 26 parents in 21 interviews.The interviews lasted for 18–53 min and were recorded. The interviews were open-ended and the first question was, “Can you tell me what you experienced when your child was born, and received cold treatment for asphyxia?” To deepen the narrations, follow-up questions were asked, “Can you tell me more? Can you give any further explanation? Can you give me an example?” Other questions asked during the interview aimed to confirm whether the researcher had understood the parents correctly.

### Ethical considerations

This study was approved by the Regional Ethical Review Board in Gothenburg, registration number 776-10. The written information that was sent home was received by the parents before the phone contact, and at the time of the interview, they were given detailed information about the purpose of this study, the voluntary nature of their participation, and about the confidentiality. As a result, all parents signed an informed consent. Since the study population was considered to be a vulnerable group, the first author had taken professional consultation, in case the interview had triggered a difficult situation for any parent. However, this was not needed in any of the situations. At the end of the meeting, the researcher stayed and discussed with the parents without tape recording.

### Data analysis

The interviews were coded from 1 to 21 and were transcribed verbatim by the first author, including non-verbal expressions, such as sighs, tears, laughter, and silence. In the five interviews in which both parents were interviewed simultaneously, the interview was transcribed and assigned different colors according to the parents’ response. The analysis was conducted primarily by AH and HW and was then reviewed by MDB, followed the steps of qualitative latent content analysis described by Graneheim and Lundman (2004). The analysis began with the repeated reading of the interview text completely to understand the context, which was followed by the identification of meaningful units. These were condensed and coded (Table I). The codes were sorted into subthemes, which were further grouped into five themes, and an overall theme (Table II).

**Table I:** Examples of meaning units, condensed meaning units and codes.

Meaning unit	Condensed meaning unit	Codes
*… in this situation, when a catastrophe like this happens, there needs to be someone who can take care of the father. I mean, one thing or another could happen …* (p. 12)	Nobody was there for the father	No one to talk to
*the doctor was very serious, it felt almost like he was preparing us for the worst, like it does not look good, it was a very serious conversation with the doctor …* (p. 20)	Serious conversation that prepares for the worst	Negative information

**Table II:** Examples of codes, subthemes, theme and the main theme.

Main theme	Overriding an emotional rollercoaster					
	
Theme	Being thrown into chaos			Being in a state of uncertainty		
Subtheme	Experiencing a traumatic delivery	Understanding that something is wrong	Feeling abandoned	Receiving information	Waiting	Not knowing
Codes	Emotional chaos	Understanding the seriousness of the situation	Alone	Continuous information	Waiting for an MRI examination.	Does not know about the NICU.
	Helpless	Must leave the operating theatre	No one to talk to	Negative information	Believes that the child is lively after the hypothermia treatment	What injuries will the child have?

## Results

The results are presented in five themes: being thrown into chaos; being in a state of unreality; being in a state of uncertainty; becoming a parent to a seriously ill child in a high-tech NICU environment; living with the experience after discharge from NICU comprehended by the overall theme–overriding an emotional rollercoaster. In the following quotations, the mother is denoted as M and the father as F. The number denotes quotations cited from the 21 different interviews.

### Overriding an emotional rollercoaster

Parents experienced that they were suddenly thrown into a chaotic situation, which often started with a traumatic delivery, followed with enduring a time of uncertainty during the infant's time in the NICU and, moreover, continued with learning successively to understand the circumstances they were set for and finally bringing the experience of having a child with birth asphyxia home, putting into words how it all affected them.

#### Being thrown into chaos

The parents understood that there was something wrong with their child during the delivery as more staff members were called, and the staff seemed busy and did not talk to them. The parents experienced the delivery as traumatic. The child was separated from the parents and was immediately transferred to the care of the staff at the NICU. Parents felt a sense of helplessness. Some of the parents witnessed a tough situation when the child was not breathing or that he/ she was born lifeless; in one case, resuscitative measure was begun before the umbilical cord had been cut.

As I was lying there, I was only thinking about whether he was alive or dead. (M18)

Immediately after the delivery, the parents did not know what they felt and how they would react; they had feelings they had never had before. The parents felt that everything was chaotic. During the caesarean section, the father was accompanied by one member of the staff from the delivery ward, but as the child was receiving neonatal care, the fathers felt that nobody focused on them. Their partner was in the recovery ward and the child was in the NICU. Since there were no staff to look after the fathers after the childbirth, this made them feel ignored and confused. They had to understand the happenings on their own.

In this situation, when a catastrophe like this happens, there needs to be someone who can take care of the father. I mean, one thing or another could happen. (F12)

#### Being in a state of unreality

All of the fathers followed their infant to the emergency room, where the NICU staff was taking care of the child. Even though the fathers experienced the neonatal emergency care as good, they also said that seeing their child receive this care was difficult. During the episode of emergency care, when the life of the infant was to be saved, the fathers lost a sense of time. The mothers who had been anesthetized were semiconscious when they had received the information from the staff. This made it all very hard to understand what was happening around them.

I could follow, ran after the uproar into a small room, where they placed him, and some doctor came and carried out the resuscitation, I don't really understand … they explained a bit about what they were doing, but I couldn't take it all in just then. (F18)

Already during the delivery, some of the mothers believed their child would be stillborn; other parents thought of their child as dead after the birth. Some parents were told by the doctor that their child would probably not survive.

Then she is brought out and is lying there, she is helpless, she is dead … She was dead … We don't know if she is going to survive the first night. (F4)

Several parents described the situation during and after the delivery as unreal. They were on autopilot, lived as if they were in a bubble, did not feel hunger, did not eat, and had trouble sleeping.

It felt unreal and I felt like a robot doing what I should. (M10)

The parents wished that everything to be fine so that their child would survive. At the same time, they could feel that it would be better if the child died, instead of being subjected to painful medical treatments or lifelong suffering with a severe handicap. Several parents said that they still, at the time of their interview, had a bad conscience about their thoughts.

A (the partner) said: ‘Couldn't she have died then’. I thought that too, that wouldn't it have been just as well. Were we meant to have a child who was not healthy? That's something that you naturally have a bad conscience about all the time. (F4)

#### Being in a state of uncertainty

All of the parents stated that they were given information that their child would receive hypothermia treatment. The parents appreciated the straightforward information given by the doctor, and they felt that they were given sufficient information on an on-going basis to the extent that the staff could offer it.

The doctor was very serious; it felt almost like he was preparing us for the worst, like it does not look good, it was a very serious conversation with the doctor. (F20)

When they were informed that their child will survive, they found themselves in a state of considerable uncertainty. They thought about the injuries the child might have received and how these would affect the child and the life of the family.

A feeling of continuously hovering between hope and despair … the whole eleven days … it was like a roller coaster. (M18)

The parents tried to prepare themselves for the eventuality that their child was injured, even if they hoped for the best, and that the child's injuries would be as minor as possible.

I prepared for her suffering from some sort of cerebral palsy, which depends quite simply on whether she was going to need a wheelchair or whether she would, to put it bluntly, be a vegetable. (F2)

The parents were informed at the start of the child's treatment period that an MRI scan of the brain would be taken to see whether the child had any brain injuries. Some parents said that they themselves had also sought more information on the Internet about cold treatment during the child's period of care and/or after the child had come home. The parents felt that they were in a state of waiting for the whole period of care. They waited for the MRI scan, and it was like waiting to receive a verdict. For many parents, it was a moment of judgment, a decision on how injured their child was.

The entire time, some kind of waiting and waiting really is the worst. (F2)

Most of the parents stated that before their child was born, they had no knowledge about NICU and what it entailed to be cared for there. To see the care environment was a shock for the parents.

Nobody had prepared me for what the neonatal section was … for someone who has never been in that world at all, it was now a shock … I wish I could have had five minutes with a nurse who just explained what the ward was. (M17)

#### Becoming a parent of a seriously ill child in a high-tech NICU environment

Facing parenthood in the NICU entailed parents adapting to the situation: becoming parents to a seriously ill child. The unfamiliar environment and not being able to hold their child prevented the parents from using their natural instincts to take care of their child. At the same time, the parents stated that the most important issue for them was that their child received the care that he/she needed to become as healthy as possible.

It was hard given that … you still wanted … to hold your little baby … even so it felt like it was worth it. (M9)

From the beginning, the parents found it very difficult to see their child connected to a technical monitoring apparatus with cables and tubes. The parents did not dare to take care of their child and felt that they were dependent on the help of the medical staff. One mother said that after a while she saw the tubes as a natural part of her child.

His lips were blue, he was pale, lifeless … the cold might be coming from below, but he could have, some cover in any event, but he had a thin, thin blanket on him … I don't know if they feel that they are cold … I presume that they feel cold like that, I'm not sure, but I felt that he was cold. (M17)

The parents explained that it was important for them that their child was calm during the hypothermia treatment. One mother repeated in her account the difficulty of her child not being calm.

It wasn't possible to keep her completely calm, even though she had received a lot of morphine … it was hard, that she was fighting it the whole time … it felt so unpleasant for her … despite the strong medicines reacting, … it was hard to see her suffering In the same way, I decided to take medicines to sleep. (M16)

Some of the mothers said that the infant had a peculiar smell during the hypothermia treatment. One mother described it as the odor of a cold suit and another mother described it as if her child smelled bad. The bad odor prevented them from enjoying contact with their child.

Many of the parents described the period of neonatal care as a step-by-step process, moving from one milestone to another in a chaotic and confused time. Every step during the NICU time was meaningful; the monitoring equipment being removed, examinations showing that the child did not have any major injuries and the child being moved to a “normal” bed instead of the incubator.

It happened so quickly, when he started recovering, every step went so very fast, you had just got used to the thought that he had been really ill. (M14)

The parents described that they were affected by seeing the situations of the other parents in the NICU. Although the parents were busy with what was happening to their child, they were aware of the difficult situations of the other parents in the unit.

There were other children there as well, so of course you thought about it and how it was going for them as well …. You start to be concerned about those around you; you understand that it's hard for them too. (F11)

The parents felt safe when they sensed that their child will not have any life-long injuries. Some felt this soon after the cold treatment, while for others it took longer time. Some of the mothers described how good it felt when they started to breastfeed their child: a sign of health. Other parents described feeling calm because of the professionalism of the staff; they trusted them and felt that they would manage even if the child were to be disabled.

I felt calm somehow because it was all so professional, unbelievably professional. (F7)

The things that they do for their child were seen by the parents as important: jumping, breastfeeding, sitting beside and talking to the child, and increasingly taking care of the child themselves. Some mothers described how important it was for them to show their child to the grandparents.

We were proud and wanted to show him … I wanted them to feel better than they did because they were also very upset because they had been very uncertain … they had built up a picture that he would look like a baby with cerebral palsy or something like that … to show that he was really fine. (M10)

The parents described their experiences of meeting or holding their child in their arms for the first time and that it was then that they felt they were parents. It was usually a positive experience, but it could also be as one mother experienced it:

Then she lay on me, gasping for air, that first meeting was really not positive at all. Then I had a real meltdown and it was as though I didn't know that I would really have her and she was thrown on me and she suffered … she just lay there so it was more of a feeling of, not holding her. I said just take her, take her she can't lie like this, I can't do it, that made me feel so much more inadequate. (M16)

The parents experienced a feeling of inadequacy when they described not being able to take care of their child themselves. They also felt that they did not know their child and they had thoughts like *is this my child?*

Then we were separated and when I came up to the neonatal clinic I felt, not even getting a slip of paper, I didn't even know that he was mine. (M17)

Several parents felt that they could not be as close to their child as they wanted to because there were no family rooms for them or they had children at home. Many parents did not want to leave the hospital grounds during the child's period of neonatal care. Some of the mothers had a bed in the maternity ward, but many felt that it was the wrong place to be.

The maternity ward is not the right environment for mothers who do not have their child with them, a neonate who is seriously ill, who you don't know if he is going to survive … it was a tragic first night, and really horrible … I was alone, I thought I was going to take my life like I'm going to jump out of the window here. (M17)

The parents experienced difficult setbacks when the condition of the child became worse. On the occasion when two mothers were holding their infants, the infants worsened in their medical state and needed support from the NICU staff. This made the mothers feel guilty of causing the infants worsened condition. Other emotionally difficult situations for the parents in the NICU were created when the staff had not informed them that the child had been moved from intensive care to intermediate care, which was another room. The parents were not prepared, and they felt that they did not decide themselves. Some of the parents believed that they could not do as they wished in the care of their child and they did not dare take the initiative when the staff did not trust them as parents.

Then I became very sad because, this was the first and only time during the stay at the hospital that I felt I could not do as I wanted … I was not allowed to hold him for some reason … it was too near the rounds. (M10)

#### Living with the experience after discharge from NICU

The parents discussed more about what happened during the child's birth and about the period of care at the NICU. The fathers did not remember everything that happened during the period of acute care and those mothers who had been anesthetized did not remember all the information that they had received. The parents felt considerable gratitude for the care that their child had received and that they had delivered their child in a hospital that had hypothermia treatment.

She would have died, she would not have had a chance, not if we had been in a small, small birth clinic, you have to be at a hospital where there are resources, if it had happened in a small hospital in a small town, she would not have survived. (F4)

The parents thought about how the hypothermia treatment had affected their child, physically and mentally. One mother wondered if the hypothermia treatment had hurt the child because her child was difficult to console during the first 2 years. Other mothers also felt that their bonding with their child had been affected by the event.

I have two completely different relationships with the two of them … I don't know, attachment, but …, I identify myself very much with N who is the eldest, she feels like a part of me, whereas with P. I still have difficulty she could just as well have been a child I was given. (M16)

Some of the parents stated that the event had left an impression on them, that up to 4 years after the child was born, they still thought about what happened during the delivery.

There is still this matter, what I still have problems with is when they said that she was dead, it is hard to get at that feeling and it was a brief feeling, but it was such a fundamental feeling that it is still here in my body. (M16)

The parents felt that the follow-up visits to the doctor after the period of care were good. They felt safe with the contact after the discharge from the hospital because it gave them the opportunity to ask the questions that they had been wondering about, and their child's development was checked.

Then we have had very good contact with the hospital afterwards as well … I felt we had preferential treatment … we could ring and visit as soon as we became worried about something. (F7)

Those parents whose child had persistent injuries felt that the follow-up provided by the medical services was not optimal. One set of parents mentioned a promised meeting with the delivery staff and the pediatrician that did not take place which made them feel forgotten. The parents wanted to be prepared ahead of any future delivery. Several mothers had received a certificate to be given a planned section to minimize the risk of experiencing an acute delivery situation again and some described how they would more question the staff's management during their next delivery. This still raised strong emotions when they visited the hospital the next time.

When we were at the hospital and were going to talk about the delivery of A. Just a planned section and I didn't believe I would react like that, but I cried as well, but that's probably normal if you come into that environment. (M5)

#### Comprehensive understanding

The experience of becoming a parent to a child with birth asphyxia can be described by the main theme of overriding an emotional rollercoaster. From being thrown into a chaotic situation, which often started with the experience of a traumatic delivery, they were to live with the experience of having a child with birth asphyxia at home. The picture of how it should be was suddenly interrupted by the traumatic delivery, the admission to the NICU and the infant being born with asphyxia, all of which elicited parents’ feeling of helplessness. At the same time, the fathers felt abandoned after delivery when the mother and child were taken in opposite directions by either the midwives or the NICU staff, and there was nobody to focus on them. As the infant needed the professional care in the NICU and hypothermia treatment, this led to an involuntary parent–infant separation in the first days of the infant's life. Beside this, the parents believed that the child would die, or already was dead. Parents’ experienced the entire situation as unreal but felt forced to go on, although they felt no hunger and it was difficult for them to sleep. Parents managed the NICU time by taking it step-by-step, experiencing different milestones in an otherwise chaotic time. During the NICU time, parents had feelings of compassion and concern for their infant. It was important that the child was calm during the hypothermia treatment. They waited for one of the most important milestones in the child's hospital stay: the MRI, where they thought they were to find out if any brain injuries and be more prepared for their future family life. Some of the parents felt themselves, even before the MRI was done, that the child was not seriously injured. After the discharge from the NICU, parents appreciated the follow-up visit to the doctor, but many of them still had concerns and unanswered questions about the delivery, the neonatal care such as the effects of hypothermia treatment. Parents felt affected by the whole experience. It affected their thoughts of having one more child and also influenced their experience of visiting the hospital the next time.

## Discussion

### Discussion of results

The experience of becoming a parent to a child with birth asphyxia treated with hypothermia at the NICU was a strenuous journey of overriding an emotional rollercoaster, that is, from being thrown into a chaotic and unexpected situation which started with a traumatic delivery to processing the difficult situation of believing the child might not survive or was to be seriously affected by the asphyxia. The results of this study also suggest that the adaption of the everyday life at home was facilitated by information from the doctor after discharge with opportunities to ask questions about the health of the child and the development. During the time in NICU, several factors hampering the parent–infant bonding could be seen in parents’ narratives; the prolonged parent–infant separation due to the hypothermia treatment, and parents’ fear of touching the child because of the high-tech equipment used for monitoring or examining the infant's health.

In this study, it seems as if the hypothermia treatment was not the focus of the parents’ experiences during the period of NICU. It was not until after discharged from the NICU that parents wondered about the effects and impact of the hypothermia treatment. This is different from Nassef et al. (2013) who found the hypothermia treatment central to the parent experience. They meant that parents experienced the warm-up after the hypothermia treatment as a “rebirth” of the child. However, in this study, we found that the traumatic delivery and the initial neonatal care, causing a separation between the mother–father–infant, were more clearly reflected in parents’ narratives. Other experiences in parents’ narratives were the fear of either loosing or having a seriously injured child, and the different milestones helping them cope during a chaotic time; the removal of monitors and technical equipment, transferring the child to a normal baby bed and having answers from the MRI.

It is notable though that parents in this study, even several years after the childbirth, could not or did not express meaningfulness in the experienced situation. This differs from research on parents’ experiences of having a premature child. For instance, Lindberg, Axelsson, and Öhrling (2008) showed that fathers of prematurely born children experienced a stronger bond with their child compared with friends who had babies born full-term. Later on, they also experienced themselves as more confident as a father. This is consistent also with findings from the United States (Black, Holditch-Davis, & Miles, 2009), where the liminality and ambiguity of the mothers decreased when there were circumstances enabling them to more independently take care of their child. Their confidence as mothers increased as the infants’ health improved, their dependence on technology decreased, and they came home from the hospital. Nassef et al. (2013) also showed the changed attitudes toward life as a result of having an infant in the NICU, alerting parents of how fragile life is and conscious of the choices they make every day. This differs from results in our study, which show that these more positive experiences were only vaguely apparent in the parent's narratives to a child with birth asphyxia. Even though parents in this study were interviewed months to years after discharge from the NICU, which differs from Nassef et al. (2013) and Lindberg et al., (2008), there are weak indications in this study that the experience of having a child with birth asphyxia was associated with significance.

Parents in the NICU are considered to be at greater risk of long-term levels of stress, worry, or tiredness which can negatively influence the parent–child interaction (Eiser, Eiser, Mayhew, & Gibson, 2005; Forcada-Guex, Borghini, Pierrehumbert, Ansermet, & Muller-Nix, 2011; Garel, Dardennes, & Blondel, 2007). Brummelte et al. (2011) and Treyvaud et al. (2011) showed that parents’ of children with neuropsychological impairments, in particular, are at a greater risk of high levels of stress after NICU. In this study, parents felt they were still strongly affected by their experience of having a child with birth asphyxia, but this was not only with parents of the three children who had persistent injuries from birth. As a possible consequence of the strong emotional crisis, parents of a child with birth asphyxia even several years after, felt it was hard to remember the time of their child's birth. They felt distressed by the thought of visiting the hospital the next time and were determined to be better prepared for any future delivery, and they would also be prepared to question the staff's management during the delivery more, if necessary.

Wigert et al. (2007) described that health-care professionals in the NICU needed further improvements to correctly deal with the emotional crisis of parents. In theory, the opportunity for the parents to share about their experiences helps them to convey their feelings and, in this way, process what has happened (Cullberg, 2006). Previous studies have shown that supportive communication strategies in the NICU as emotional responsiveness to parents’ needs (Fenwick, Barclay, & Schmied, 2001; Mok & Leung, 2006) and information and knowledge about the infant's care (Arockiasamy et al., 2008; Heermann et al., 2005; Wigert et al., 2006) are helping parents to cope more easily. This is consistent with our results. Parents in this study were satisfied when they had honest and frequent information from the NICU doctor and when they believed that nurses gave them possible answers to their questions. However, in contrast to Nassef et al. (2013) the results of this study describe the importance of talking and having information from the NICU doctor also after discharge. It also gives perspectives to questions about possible lack of the support after discharge from NICU.

Parents in this study expressed that they felt safer when they were thoroughly informed and had the opportunities to ask questions about their child's health and development during neonatal care as well as after discharge from the NICU. Also, the lacking information from health-care professionals after discharge was a reason for being dissatisfied with the care received. In a study by Kowalski, Leef, Mackley, Spear, and Paul (2006) about communication in the NICU, parent–doctor and parent–nurse conversations were infrequently close to the family's discharge. According to Ziegert (2011) when everyday life changes for the family in the event of chronic illness or disability, contact between the family and professional caregivers is essential at different times of the event. Altogether this may indicate the importance of available support for the family where the child was born with birth asphyxia, not only during the NICU time but also after discharge.

The results in this study also indicate that there are some circumstances for the parents of infants with birth asphyxia that may hamper the parent–infant bonding process. One of the factors is associated with the high-tech environment and high-tech equipments used in the treatment of the infant with birth asphyxia. Technical apparatus connected to the infant's body contributes to parents’ fear of touching the infant, and at the same time physical contact and closeness between parent and infant are essential for the parent–infant bonding process (Cleveland, 2008; Hall, 2005; Jämsä & Jämsä, 1998; Wigert, Hellström, & Berg, 2008). As a prolonged problem of physical closeness between parent and infant, family rooms in the NICU could not be offered to all of the parents in this study, although research show that physical possibility to being close to the infant in the NICU round-the-clock, facilitates the parent–infant bonding (Lagercrantz, Hellström-Westas, & Norman, 2008). Mothers in this study clearly narrate about their feelings of involuntarily being separated from their infant. They also experienced the maternity ward as the wrong place for them and described it as painful since they saw all healthy newborns together with their parents. In contrast to family rooms, this kind of experience makes it more difficult to perceive yourself as the mother of your child (Callery, 2002; Wigert et al., 2006; Wigert et al., 2008).

During the hypothermia treatment, the infant receives pain medication and sedation, which makes it difficult to interpret the signals of the infant. According to Bowlby (1969) this may hamper the parent–infant bonding. In this study, some mothers several years after the neonatal care, still wonder if the mother–infant relationship was influenced by the separation between them. One mother even explained that she did not know whether it was her child or not that she saw in the NICU. This is in agreement with previous studies which show that parents in the NICU, may experience ambivalence to the child several years after discharge (Jackson et al., 2003; Wigert et al., 2006). Some of the mothers in this study felt that they were not in charge of their infant, but instead were dependent on the NICU staff to take care of their child. The fact that mothers comply with the instructions of the staff has been described in other studies (Callery, 2002; Conz, Merighi, & Jesus, 2009; Heermann et al., 2005) and Fenwick et al. (2001) even named this as a struggle to be a mother.

In Family Centered Care (FCC), parents are considered to be a partner in care. Parents are significantly involved in the decision making and the care of the child. The care is not planned according to the child itself, but to the entire family and family members (Harrison 1993; Jolley & Shields, 2009). According to the principles of FCC, described by Harrison (1993), this study shows some drawbacks in the FCC given, which is exemplified when the fathers during the neonatal emergency care experienced themselves ignored by the staff from the delivery who took care of the mothers, and the NICU staff who focused on the care of the infant.

Nassef et al. (2013) found that nurses play a vital role in providing individual support to parents of infants with birth asphyxia. The results of this study suggest that nurses should support parents by creating and strengthening the different milestones that parents seem to need during the chaotic NICU time. This could be done by clarifying steps in the infant's recovery and promoting parent–infant contact that we now know is important for the mother of a child with birth asphyxia, as the breastfeeding event. This suggestion can be underlined by a study by Palmér, Carlsson, Mollberg, and Nyström (2010), which showed that when the mother has problems in breastfeeding the child, it may negatively affect the mothers’ well-being and also the experience of being a mother. According to Nassef et al. (2013), the parent–infant bonding can be promoted also by gently taking care of the moment after rewarming, since the infant is then more awake, starts to move, and starts to behave as a normal newborn.

### Methodological considerations

In qualitative research, the concepts such as credibility, dependability, and transferability have been used to describe various aspects of trustworthiness. Questions regarding the credibility refer to the focus of the study and the selection of context, participant, and approach to gather data (Graneheim & Lundman, 2004). The results of this study are strengthened by the choice of open-ended interviews, since they in a sparsely researched area may increase the possibilities of more data (Polit & Beck, 2012). Moreover, the majority of the Swedish speak families that were available to contact participated in the study. However, there is always a risk that important data are lost when some of the parents refuse participation. To increase the transferability of the study results, we, while preserving the participants’ anonymity, also presented several variables. There was, for example, a variety and heterogeneity in the sample associated with sex, parental age, first-time parents, delivery, and the NICU experience—fruitful for a trustworthy result. At the same time, qualitative research is always a product of its context, which hampers generalizations of the results. The presented sample makes it easier for the reader to decide whether or not the findings are transferable to another context. This is also strengthened by many quotations of the interviews.

The choice of latent content analysis is a marked way of expressing the level of interpretation of the data. Meanwhile, as qualitative latent content analysis produces “data-near” results, interpretations are always made by the researcher (Sandelowski, 2010). The credibility of the results is also strengthened by the two researchers analyzing and discussing the choice of meaning units and relationships between the parts and the whole of the texts. The three researchers involved in this study also strengthen the dependability, since it decreases the risk that data and interpretation of data changes over time. However, from another perspective, the latent content analysis as a method sets the limit at a lower level of interpretation, which may lead to a limited picture of the deeper experience of becoming a parent to a child with birth asphyxia (Dahlberg, Dahlberg, & Nyström, 2008).

The weakness of this study can be the different means of interviewing parents, since some of them were interviewed one by one and some of them as a couple. There may be a risk of interference in each other's narrations. This was counteracted by coloring mothers and fathers narratives differently in the transcribed texts, and in doing so, we could see that no obvious interference could be discovered. Instead, the parents’ choice of interview context seemed to support openness to the interviewer and parents’ narratives seemed to grow together (Dahlberg et al., 2008).

## Conclusion and implication for practice

This study suggests that the experience of becoming a parent to a child with birth asphyxia treated with hypothermia at the NICU is a journey of overriding an emotional rollercoaster. The journey seems to be primarily affected by the experience of being thrown into a chaotic and unexpected situation, which began with a traumatic delivery and an abrupt admission of the infant to the NICU, rather than the hypothermia treatment itself. However, the hypothermia treatment prolonged the physical parent–infant separation and in combination with the high-tech equipment monitoring the infant's health, this seems to affect the parent–infant bonding. The difficult situation of believing the child might not survive or was to be seriously injured by the asphyxia, underlines the importance of information and emotional support during and after the NICU discharge, especially from the doctor. Nursing support should be better based on the principles of FCC, considering the needs of the entire family and its members. To support the family, the NICU staff should be present for the fathers during the emergency care. Whenever possible, the nursing support should be focused on explaining and clarifying steps in the infant's recovery to create milestones that help parents to cope up during a chaotic time. The health-care system and the health-care professionals should continuously strengthen the parent–infant bonding by creating possibilities for physical closeness between them, as in giving parents possibilities for feeding the infant, touching the infant, or offering family rooms to the parents in the NICU. Further research is needed to answer questions about how the experience of having a child with birth asphyxia affects the family planning or socioeconomic circumstances, and how the most effective support during delivery and hospital stay in NICU, but also after discharge from the NICU should be designed.
